# Effect of marigold (*Tagetes erecta* L.) on soil microbial communities in continuously cropped tobacco fields

**DOI:** 10.1038/s41598-022-23517-x

**Published:** 2022-11-16

**Authors:** Feiyan Huang, Xiaopeng Deng, Lingling Gao, Xianjie Cai, Ding Yan, Yongzhan Cai, Xiaolong Chen, Min Yang, Wenjie Tong, Lei Yu

**Affiliations:** 1 Kunming University/Yunnan Urban Agricultural Engineering & Technological Research Center, Kunming, 650214 China; 2 Yunnan Academy of Tobacco Agricultural Sciences, Kunming, 650021 China; 3 Center of Agricultural Products Processing, Henan Academy of Agricultural Sciences, Zhengzhou, 450002 China; 4 Material Procurement Center, Shanghai Tobacco Group Co., Ltd., Shanghai, 200082 China; 5 Yunnan Provincial Tobacco Company Qujing Branch, Qujing, 655000 China; 6 Tobacco Leaf Purchase Center, China Tobacco Henan Industrial Co., Ltd, Zhengzhou, 450000 China

**Keywords:** Ecology, Microbiology, Plant sciences

## Abstract

Root-knot nematode disease is a catastrophic soil-borne disease in tobacco production. The regulation of natural microbial communities is considered a good disease management approach to suppress the incidence of soilborne diseases. In this study, the effects of tobacco (*Nicotiana tabacum* L.)-marigold (*Tagetes erecta* L.) rotation on the diversity and structure of soil microbial communities in continuously cropped tobacco fields were analyzed to manage this devastating pathogen. The results showed that the soil bacterial OTUs increased after marigold rotation and that the bacterial Shannon, ACE, Chao1 index, and fungal Shannon index were higher in the tobacco-marigold rotation fields than in the continuously cropped tobacco fields by 3.98%, 10.37%, 5.46%, and 3.43%, respectively. After marigold rotation, the relative abundances of Actinobacteria, Acidobacteria, and Ascomycota increased by 28.62%, 107.50%, and 57.44%, respectively, and the proportion of beneficial bacterial genera such as *Nocardioides*, *Gemmatimonas*, and *Bradyrhizobium* increased. In addition, our results also showed that rotation of marigold could effectively reduce the incidence of root-knot nematodes in the next crop of tobacco. These results indicate that marigold rotation had a positive effect on the soil microecological environment of continuously cropped tobacco fields, reducing the obstacles to continuous cropping of tobacco.

## Introduction

Root-knot nematode (RKN) disease caused by *Meloidogyne* spp. is a serious threat to tobacco, affecting production worldwide, including China. This disease is very common in tobacco producing areas of China. The affected area in Yunnan Province alone is more than 26,000 hectares, causing yield losses of 30 ~ 50%^1^. Due to the area of arable land, topography, and landforms in Yunnan, continuous cropping is the main planting pattern for tobacco in this province, which will undoubtedly aggravate the occurrence of tobacco soil-borne diseases such as root-knot nematode disease^2^. In recent years, plant rhizosphere has attracted much attention as a hot spot ecological environment for plant-microorganisms interaction^3^. The regulation of natural microbial communities is considered to be one of the most promising strategies for improving soil health to achieve integrated and sustainable disease management^4^. For example, the diversity of plant species in the ecosystem is maintained through crop rotation and intercropping, so as to prevent plants from biological stress by improving the diversity and structure of rhizosphere microorganisms^5,6^. Moreover, biological control protects plants from soil-borne pathogens through potent antagonistic microbes, inducing host resistance, niche exclusion and direct antagonism^7^.

Marigold (*Tagetes erecta* L.) is a species in the family Asteraceae. It is commonly grown as an ornamental plant in many regions of the world, and is famous for its medicinal and antibacterial properties^8^. *Tagetes* species can produce allelopathy compounds with antimicrobial activity, which exhibit cytotoxicity to plant nematodes^9,10^, and can antagonist 14 genera of plant-parasitic nematodes^11^. Xu et al. identified 126 substances in *Tagetes* plants^12^. Among them, the thiophenes (including 3-buten-1-ynyl-2,2′-bithienyl [BBT, I] and α-terthienyl [α-T, II]) in the roots of marigold are considered the main active substances that are cytotoxic to nematodes^13,14^. Although the ecological functions of these compounds have not been clarified, past studies have reported that they have biological activities against plant pathogens and nematodes^15,16^. There are many reports on the successful control of crop diseases by marigold. For example, rotating or intercropping marigold with crops susceptible to nematodes can effectively reduce the number of nematodes in the soil and increase the diversity index of nematodes, which is conducive to the balance of nematode community structure^17,18^. Marigold rotation, intercropping and mulching could effectively inhibit nematodes infection in eggplant, tomato and soybean^19,20^. The rotation of Chinese cabbage and marigold can significantly reduce the incidence of clubfoot disease in the next crop^21^. In addition, marigold can also impact soil microbial communities: intercropping marigold with tobacco can increase the diversity of rhizosphere soil bacterial communities^22^. A marigold-angelica rotation had a substantial impact on the composition of soil fungal communities^23^.

In general, different marigold utilization methods can play a demonstrably positive role in regulating soil microecology. However, due to the broad-spectrum biological activity of marigold root exudates, the work on the impact of different utilization methods of marigold on soil microbes is still limited^8,24^. Therefore, we hypothesized that tobacco-marigold rotation could effectively control the incidence of tobacco root-knot nematodes and alleviate the obstacles of tobacco continuous cropping. In order to explore the microbiological mechanism that occurred under tobacco-marigold rotation, the diversity and community composition of rhizosphere bacteria and fungi were analyzed by high-throughput sequencing. The results of this study will assist in developing an effective and sustainable agricultural rotation pattern to mitigate the incidence of tobacco root-knot nematodes.

## Materials and methods

### Field experiment design and description

**Figure 1:**
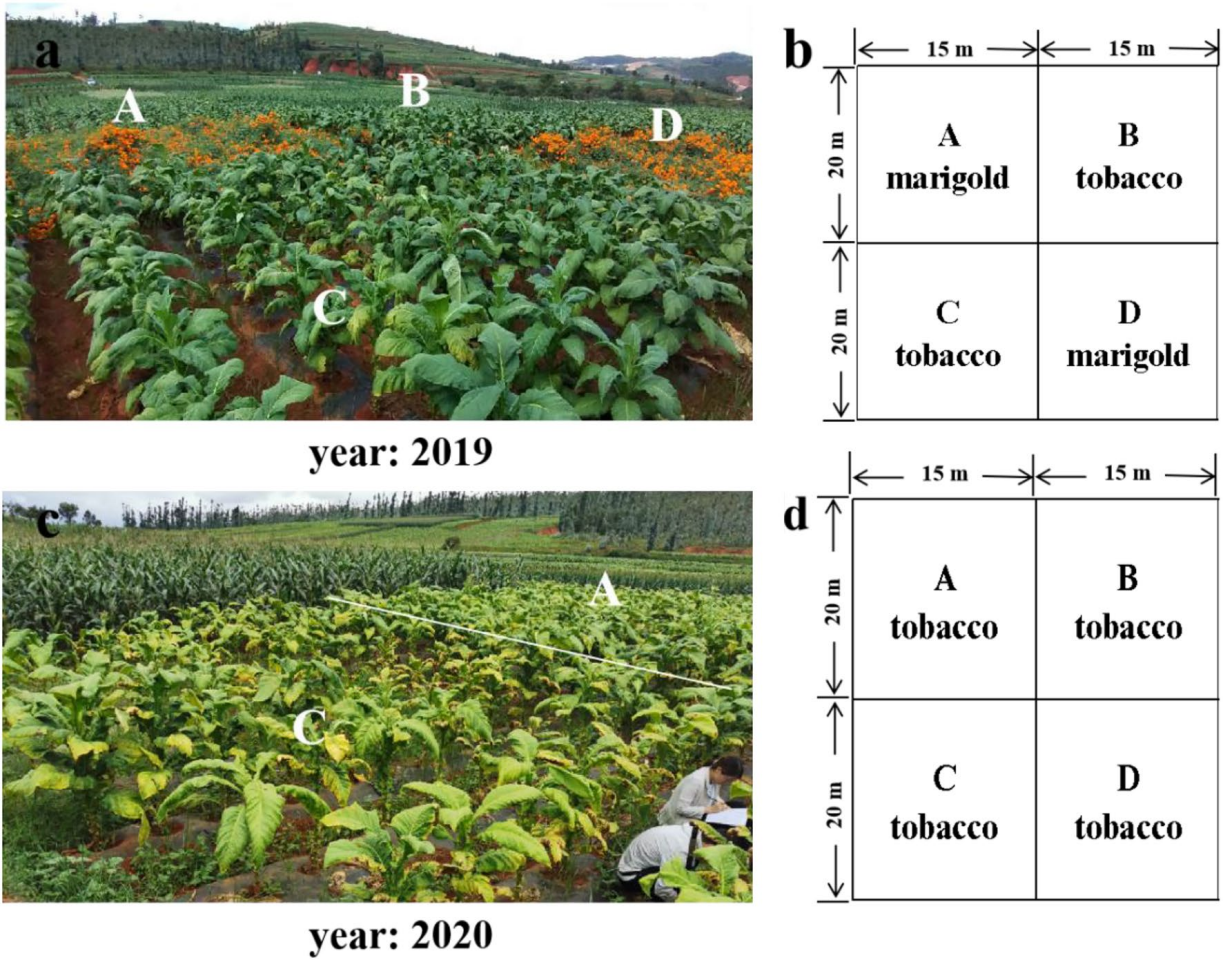
Plot design for microbial communities analysis of the plant rhizosphere. (**a**) 2019 field trial planting map; (**b**) the plot test design of tobacco-marigold rotation in 2019; (**c**) 2020 field trial planting map; (**d**) the plot test design of marigold-tobacco rotation in 2020.

The field test was conducted from March 2018 to September 2020. The experimental plot was located at Gaocang Street (24°30′N, 103°32′E), Hongta District, Yuxi City, Yunnan Province, at an altitude of 1760 m. The soil was red loam. Tobacco had been planted continuously in this plot for 5 years, and the tobacco variety was K326. In 2018, we did not carry out any treatment, and the whole experimental site continued to grow tobacco. According to the observation of typical root-knot nematodes symptoms, the incidence of root knot nematode disease in this experimental site was 63.3%. In 2019, we divided the experimental site into four plots. Marigold was planted in plots A and D, and tobacco continued to be planted in plots B and C (Fig. 1a,b). In 2020, tobacco was planted in four plots A, B, C and D (Fig. 1c,d). Conventional single-ridge planting was adopted for both tobacco and marigold. The row spacing and plant spacing were 1.2 m × 0.6 m for tobacco and 0.4 m × 0.4 m for marigold.

### Soil sample collection

During the vigorous growth period of marigold and tobacco, the rhizosphere soil of marigold and tobacco in the 0–20 cm plow layer was collected. Approximately 500 g of soil was collected using the 5-point sampling method. Sampling was repeated three times, after removing the impurities and residual fine roots, the soil samples were put separately into sterile self-sealed bags, and the samples were immediately transported to the laboratory in liquid nitrogen and stored at – 80 °C for later use. The test included two treatments: a. treatment T, which was continuous tobacco cropping (Fig. 1a,b: plots B and C); b. treatment C, which was tobacco-marigold rotation, i.e., planting tobacco in 2018 and marigold in 2019 (Fig. 1a,b: plots A and D).

### DNA extraction and polymerase chain reaction amplification (PCR)

Total DNA in soil samples was extracted in accordance with the instructions of a FastDNA SPIN Kit for Soil (MP Biomedicals, CA, USA). DNA concentration and purity were detected using a NanoDrop2000, and the DNA quality was determined using 1% agarose gel electrophoresis.

Primers ITS1 F (5'-ACTTGGTCATTTAGGAAGTAA-3') and ITS2 R (5’-BGCTGCGTTCTTCATCGATGC-3') were used to amplify the fungal 18S rRNA gene. The amplification system was as follows: 4 μL 5 × FastPfu buffer, 2 μL 2.5 mmol/L dNTPs, 0.8 μL forward primer (5 μmol/L), 0.8 μL reverse primer (5 μmol/L), 0.4 μL FastPfu polymerase, 0.2 μL BSA, 10 ng DNA template and water added ddH_2_O to a final 20-μL volume. The amplification procedure was as follows: pre-denaturation at 95 °C for 3 min, 36 cycles of denaturation at 95 °C for 30 s, annealing at 55 °C for 30 s and extension at 72 °C for 45 s, followed by a final extension at 72 °C for 10 min. Primers 338F (5'-ACTCCTACGGGAGGCAGCAG-3') and 806R (5'-GGACTACHVGGGTWTCTAAT -3') were used to amplify the V3-V4 region of the 16S rDNA. All the conditions of this PCR step were the same as those of the 18S rRNA PCR amplification except that only 27 cycles of thermal cycling were performed. The PCR products were recovered from 2% agarose gel and were further purified using the AxyPrep DNA Gel Extraction Kit (Axygen Biosciences, CA, USA) according to the manufacturer’s instructions. The PCR amplicons were sequenced on the Illumina MiSeq PE300 platform in Shanghai Majorbio Bio-Pharm Technology Co., Ltd.

### Sequence quality control and analysis

Trimmomatic software was used for quality control of the original sequencing sequences, and FLASH software (version 1.2.11, https://ccb.jhu.edu/software/FLASH/index.shtml) was used for sequence assembly^25^. Usearch software^26^ (version 11, http://www.drive5.com/usearch/) was used to filter the obtained sequences and remove the chimera sequences to obtain the valid sequences. Uparse software was used to divide the operational taxonomic units (OTUs) at a similarity level of 97%. The RDP classifier software^27^ (version 2.13, https://sourceforge.net/projects/rdp-classifier/) and the SILVA database^28^ (version 138, http://www.arb-silva.de) were used for species annotation. Mothur (version 1.30.2, https://www.mothur.org/wiki/Download_mothur) was used to generate the dilution curves, to calculate the library coverage and the Shannon, Simpson, abundance-based coverage estimator (ACE), and Chao1 indices, and to evaluate the species diversity and abundance indices. Principal coordinate analysis (PCoA) was performed using the Bray–Curtis distance algorithm established by QIIME software (version 1.9.1, http://qiime.org/install/index.html). All raw data related to 16S and 18S amplicon sequences can be found in supplementary material (Supplementary File 1–24).

### Data analyses

The differences in the microbial diversity index were compared using Analysis of Variance (ANOVA, IBM SPSS 22.0). Significance was determined at the P < 0.05 level and at the 95% confidence level by Duncan’s test.

## Results and analysis

### Alpha diversity analysis and comparison of differences in OTUs

**Figure 2:**
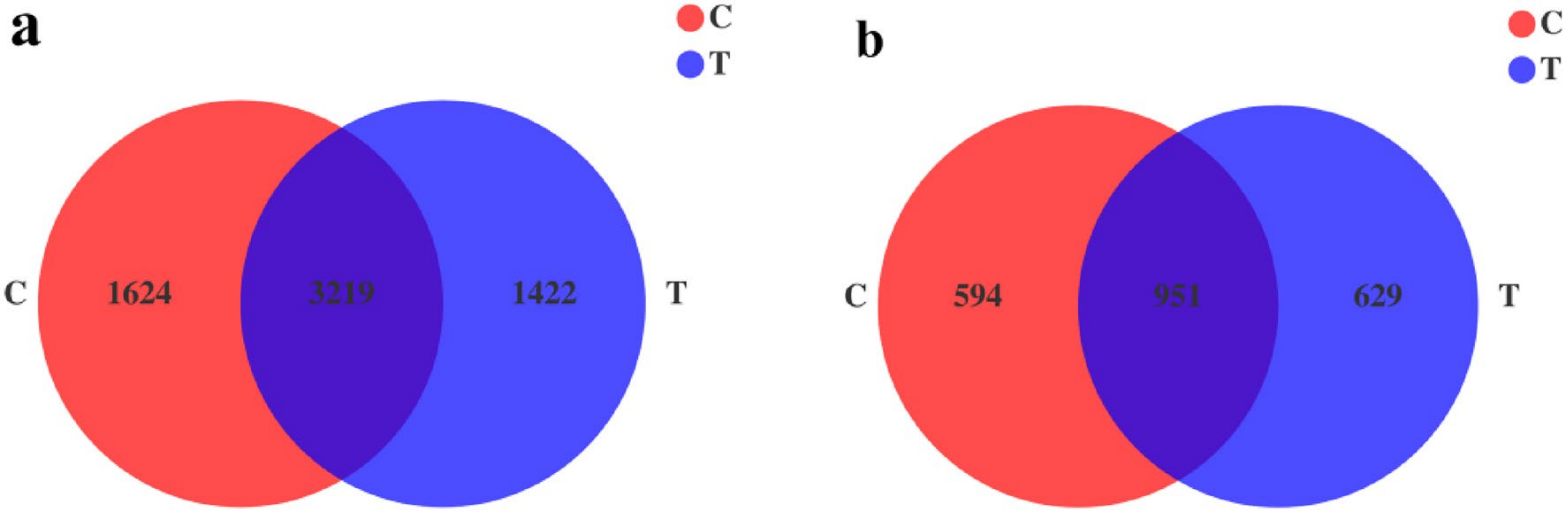
A Venn diagram shows the number of unique, shared, and common operational taxonomic units. (**a**) bacterial, (**b**) fungal.

Table 1 shows the OTU abundance and alpha diversity index of bacterial and fungal communities in the rhizosphere soil samples of different treatments. A total of 6265 bacterial OTUs and 2174 fungal OTUs were obtained from the two treatments. The bacterial OTU abundance was higher in treatment C than in treatment T, and the number of OTUs unique to treatment C was 1.14 times the number unique to treatment T. However, the fungal OTU abundance was not significantly different between the two treatments (Fig. 2).

**Table 1:** Bacterial and fungal Alpha-diversity index of diferent treatments.

Treatments		OTU abundance	Shannon	Simpson	ACE	Chao1	Coverage/%
Bacteria	C	4843	6.53	0.0054	4861.10	4691.56	97.47%
	T	4641	6.28	0.0105	4404.23	4448.55	97.50%
Fungal	C	1545	4.82	0.0195	1079.03	1070.55	99.71%
	T	1580	4.66	0.0260	1166.22	1167.60	99.62%

The alpha diversities of bacteria and fungi shows a certain difference between the two treatments. The Shannon, ACE and Chao1 index of bacteria in treatment C were 3.98%, 10.37%, and 5.46% higher, respectively, than those in treatment T, indicating that the diversity and richness of rhizosphere bacterial communities were higher in treatment C than in treatment T. The fungal Shannon index of treatment C was also 3.43% higher than that of treatment T, but the ACE and Chao1 index of treatment C were 7.48% and 8.31% lower, respectively, than those of treatment T.

### Community species and relative abundance of bacterial and fungal

**Figure 3:**
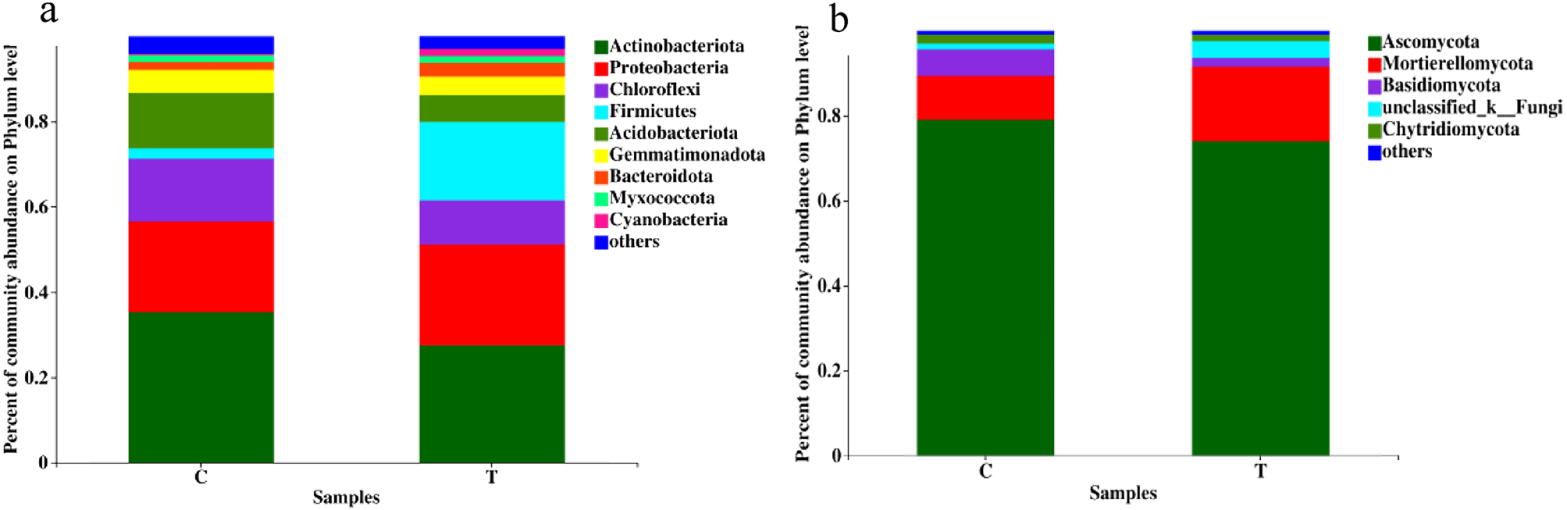
Bacterial (**a**) and fungal (**b**) community composition on Phylum level at different treatments.

**Figure 4:**
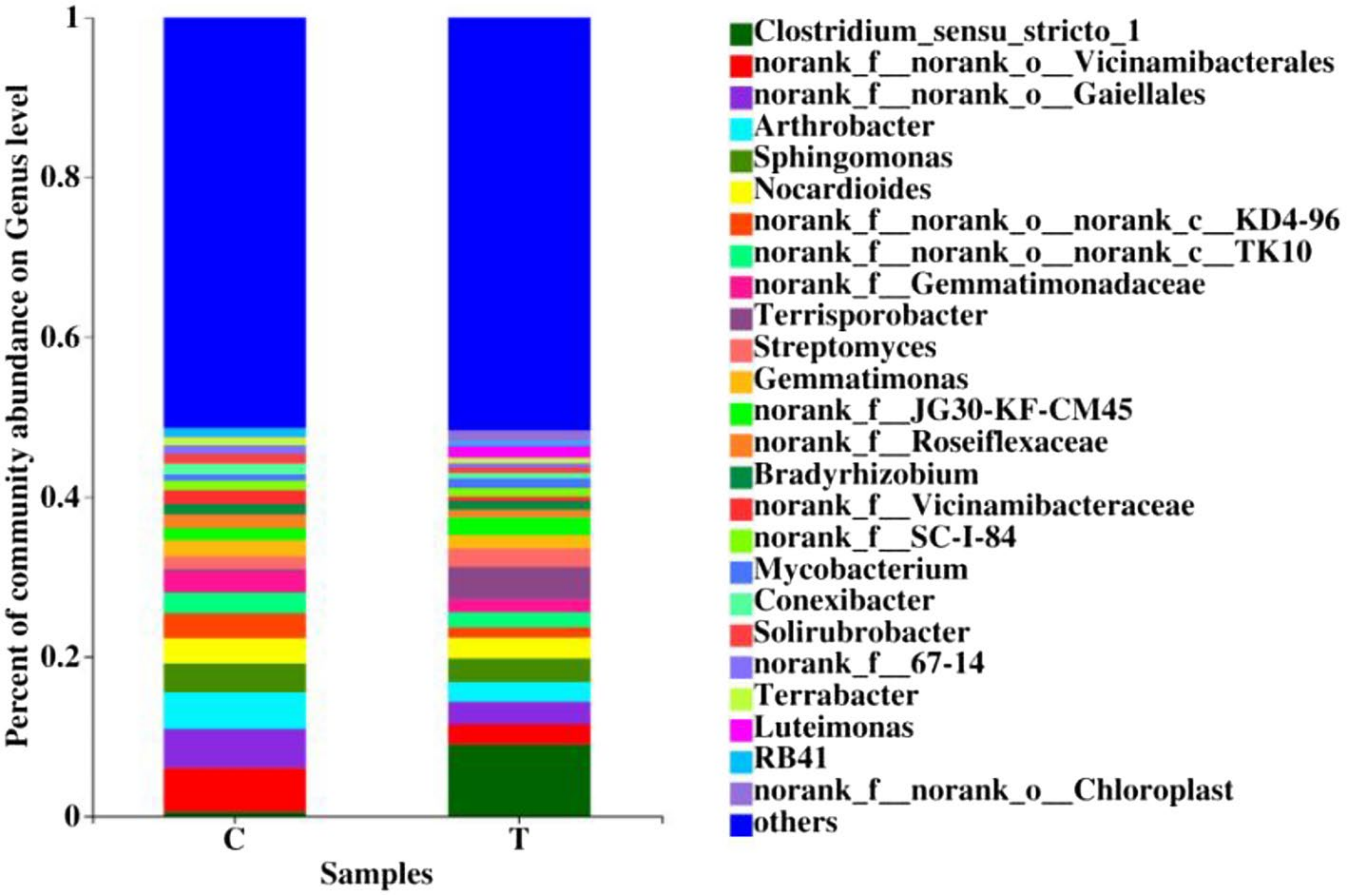
Bacterial community composition on genus level at different treatments.

**Figure 5:**
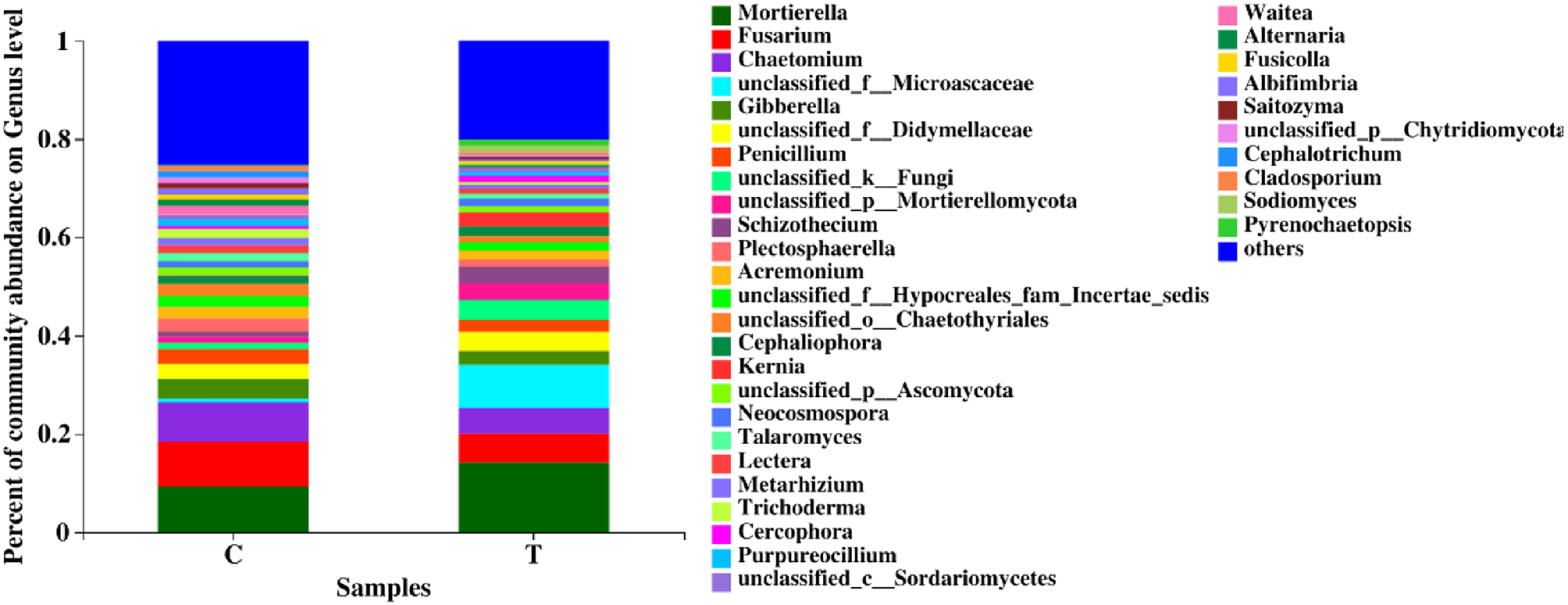
Fungal community composition on genus level at different treatments.

Figures 3, 4 and 5 show the relative abundances of bacterial and fungal phylum and genus at the 97% clustering level based on the species annotation results of the representative sequences in each OTU. The bacterial flora composition at the phylum level was different between the two treatments (Fig. 3a). The bacterial of treatment C at the phylum level was mainly composed of Actinobacteria (35.37%), Proteobacteria (21.17%), and Chloroflexi (14.75%). The bacterial of treatment T at the phylum level was mainly composed of Actinobacteria (27.50%), Proteobacteria (23.59%), and Firmicutes (18.40%). The relative abundances of Actinobacteria and Acidobacteria were higher in treatment C than in treatment T by 28.62% and 107.50%, respectively, whereas the relative abundances of Proteobacteria and Firmicutes were lower in treatment C than in treatment T by 10.26% and 86.74%, respectively. At the genus level (Fig. 4), the relative abundances of *Arthrobacter*, *Sphingomonas*, *Nocardioides*, *Gemmatimonas*, and *Bradyrhizobium* were higher in treatment C than in treatment T by 88.16%, 20.13%, 22.96%, 23.67%, and 16.07%, respectively, whereas the relative abundance of *Streptomyces* was 29.87% lower in treatment C than in treatment T.

The fungal of the two treatments at the phylum level were both mainly composed of Ascomycota and Mortierellomycota, which accounted for more than 85% of the total microbiota (Fig. 3b). The relative abundance of Mortierellomycota was lower in treatment C than in treatment T, whereas the relative abundances of Ascomycota, Basidiomycota, and Chytridiomycota were all higher in treatment C than in treatment T. At the genus level (Fig. 5), the relative abundances of *Fusarium*, *Chaetomium*, and *Penicillium* were all notably higher in treatment C than in treatment T by 57.44%, 52.84%, and 24.79%, respectively, whereas the relative abundance of *Mortierella* was 35.40% lower in treatment C than in treatment T.

### Principal coordinates analysis (PCoA) clustering and hierarchical clustering of bacterial and fungal community compositions

**Figure 6:**
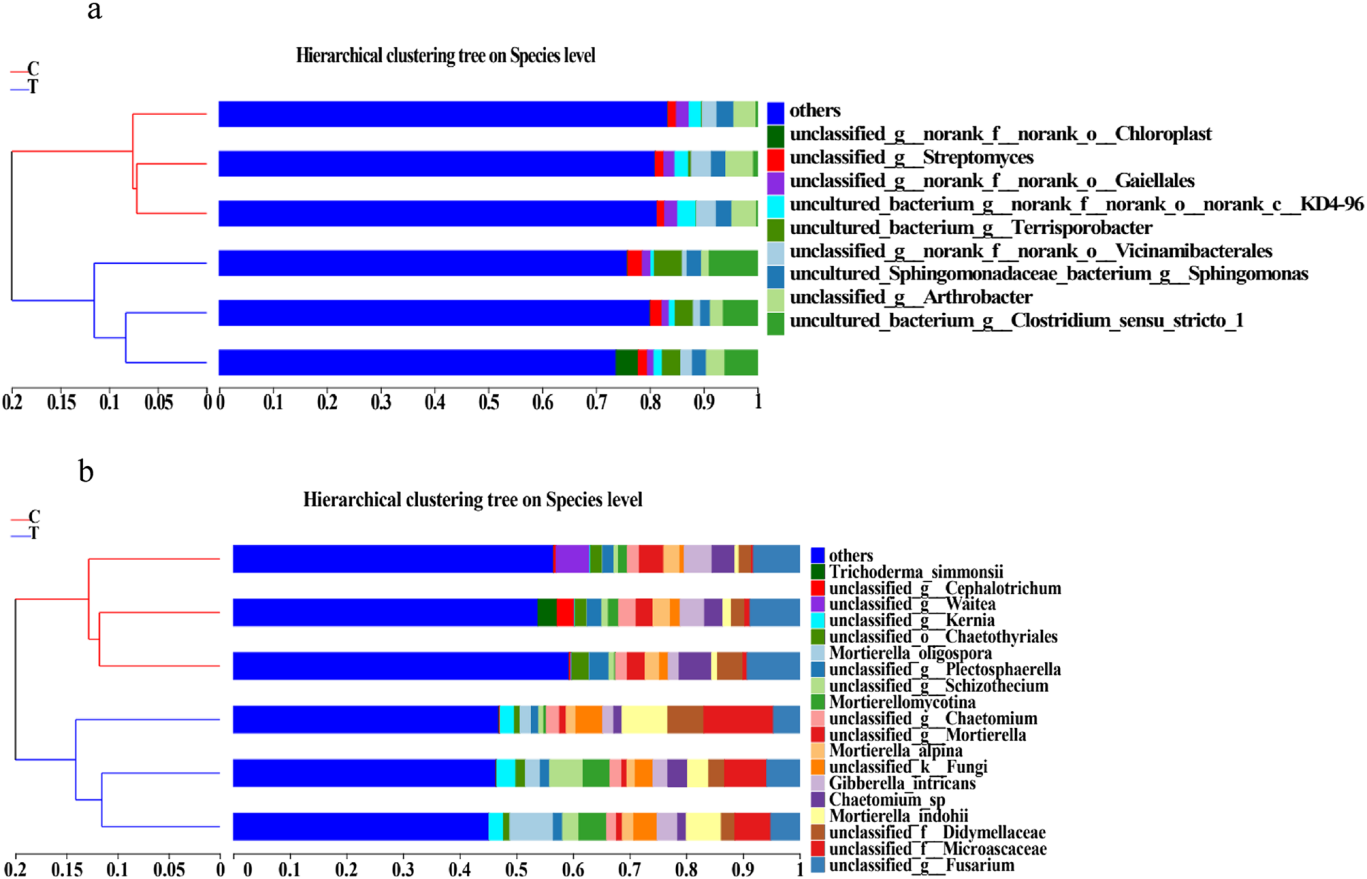
Hierarchical clustering analysis showing the changes in the composition of bacterial (**a**) and fungal (**b**) communities.

Hierarchical clustering was performed on each sample at the species level (Fig. 6). The abundances of bacterial and fungal species from treatments C and T each clustered into two branches, indicating that the abundances of bacterial and fungal species changed significantly after marigold rotation.

**Figure 7:**
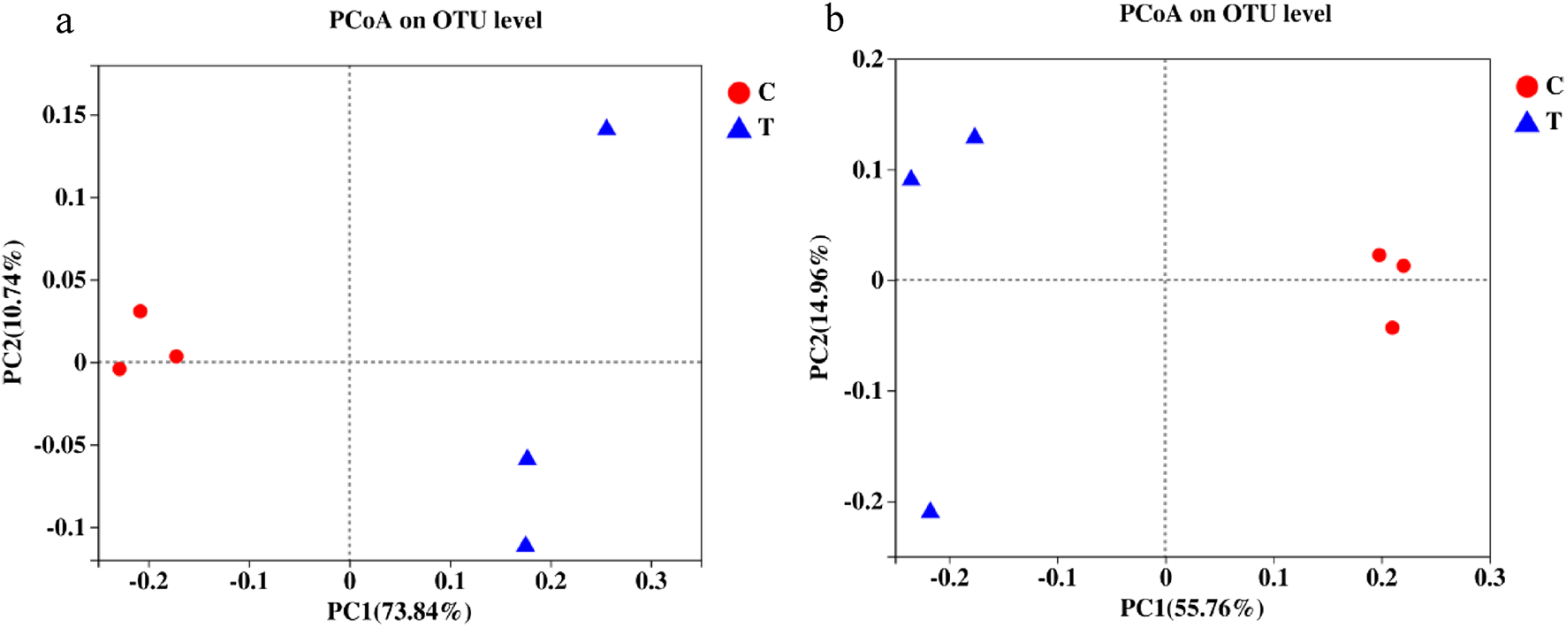
Principal coordinate analysis (PCoA) plots based on the Bray–Curtis dissimilarity matrix showing the changes in the structure of bacterial (**a**) and fungal (**b**) communities.

A PCoA was performed using the Bray–Curtis distance algorithm (Fig. 7). The results showed that the bacterial and fungal community compositions at the OTU level were significantly different between treatments C and T. The bacterial community of treatment C was mainly distributed on the left side of the PC1 axis, whereas the bacterial community of treatment T was mainly distributed on the right side of the PC1 axis; the distance between the two treatments was relatively large, indicating that the bacterial community compositions of the two treatments were considerably different (Fig. 7a). In contrast, the fungal community showed that treatment C was distributed on the right side of the PC1 axis, and treatment T was distributed on the left side of the PC1 axis (Fig. 7b), but the fungal community compositions of treatments C and T were markedly different. The above results indicate that marigold rotation greatly affects the distribution and composition of soil microbial communities in continuously cropped tobacco fields.

### Effect of marigold rotation on the incidence of root-knot nematodes

**Figure 8:**
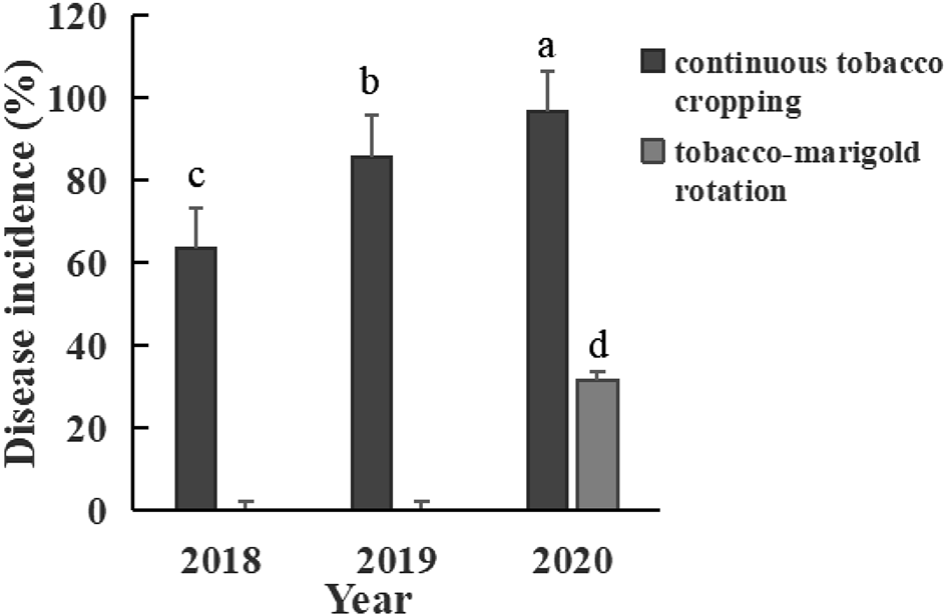
Effect of marigold crop rotation on the incidence of root-knot nematodes disease at fields.

In order to further evaluate the impact of marigold rotation on soil health of continuous cropping tobacco fields, we calculated the incidence of root-knot nematodes in marigold rotation plots and continuous tobacco cropping plots respectively. As shown in Fig. 8, the incidence of the whole experimental site in 2018 was 63.3%. In 2019, the incidence of two tobacco continuous cropping areas (plots B and C) was 85.7%. In 2020, the incidence of two tobacco continuous cropping areas (plots B and C) rose to 96.7%, which was 33.4% and 11.0% higher than that in 2018 and 2019 respectively. But it is worth noting that the incidence in areas A and D decreased to 31.6% after rotation of marigold for one year, which was 65.1% lower than that in areas B and C (96.7%) after continuous cropping for 3 years. The results suggest that marigold rotation could effectively reduce the incidence of root-knot nematodes in the next crop of tobacco.

## Discussion

Among the many factors that cause obstacles to continuous cropping of flue-cured tobacco, the imbalance of rhizosphere microecology is the main factor. Soil microbes are the most biologically active component of the soil microecosystem and play an important role in the occurrence of soil-borne diseases^29,30^. The loss of soil microbial diversity can easily lead to an increase in plant soil-borne diseases^31^. High microbial diversity and activity are conducive to promoting plant growth, enhancing plant defenses, and inhibiting the occurrence of soil-borne diseases^32,33^. In this study, the soil bacterial OTU abundance increased after marigold rotation, and the bacterial Shannon, ACE, Chao1 index, and fungal Shannon index were all higher in the tobacco-marigold rotation fields than in the continuously cropped tobacco fields, indicating that tobacco-marigold rotation improved soil microbial diversity and richness. In general, the higher the alpha diversity index of the community, the more complex and stable the microbial community structure. There may be two reasons for this phenomenon: first, crop rotation is conducive to the reproduction of soil microbes, which increases the number of microbes, enhances metabolic capacity, and maintains high microbial activity and diversity in the soil; second, the root exudates greatly affect soil chemical properties, and changes in soil chemical properties indirectly impact the distribution of soil microbial communities^34^.

Past research has shown that soil microbes respond quickly to changes in the external environment such as planting patterns, resulting in dynamic changes in microbial abundance and composition^35^. In this study, the different microbial distributions between samples of the two treatments indicate significant differences in the soil bacterial and fungal communities of the tobacco-marigold rotation fields and the continuously cropped tobacco fields. First, the relative abundances of Actinobacteria and Acidobacteria in the soil were significantly higher in the tobacco-marigold rotation fields than in the continuously cropped tobacco fields. Acidobacteria are mainly involved in the iron cycle and single-carbon compound metabolism and play an important role in the degradation of plant residues^36^. Actinobacteria can degrade cellulose, lignin, and lignocellulose by secreting hydrolase enzymes such as β-glucosidase and xylanase, thereby utilizing refractory carbon sources, and their relative abundance often increases with increasing dissolved organic carbon^37,38^. In addition, the relative abundances of *Nocardioides* (which is related to plant growth and biological protection), *Gemmatimonas* (which is involved in phosphorus metabolism), and *Bradyrhizobium* (which can decompose organic matter and promote the soil carbon cycle and nitrogen fixation) showed increasing trends after marigold rotation.

Compared with the continuous cropping of tobacco, the relative abundance of Ascomycota in the soil was significantly higher after marigold rotation. Previous studies have shown that an increase in ascomycetes in the soil is conducive to the decomposition of plant residues. In fact, saprophytic ascomycetes are considered the main fungal group involved in the initial step of plant litter decomposition, i.e., the decomposition of soluble components and total cellulose. Their growth rate is positively correlated with the addition of fresh litter and available nitrogen^39,40^. We also found that the relative abundance of *Penicillium* significantly increased after marigold rotation. The increase in the abundance of *Penicillium* in the soil is conducive to the health of the soil environment because *Penicillium* plays an important role in the secretion of cellulase and antibiotics^41^. The increases in the relative abundances of these beneficial bacteria and fungi in the soil after marigold rotation has a positive effect on the soil micro-ecological environment. Most notably, our results also showed that rotation of marigold could effectively reduce the incidence of root-knot nematodes in the next crop of tobacco (decreased 65.1%). However, there are still significant limitations to explore the changes of microbial community only through 16S and 18S high-throughput sequencing. In the future, we will combine the traditional isolation and culture, metagenomics methods to comprehensively analyze the microbial community function after marigold-tobacco rotation.

## Conclusions

This study analyzed the role of tobacco-marigold rotation in regulating the soil microbial community structure under continuous cropping and provides a new idea for sustainable agriculture for successful prevention control of tobacco root-knot nematodes disease. Marigold rotation can increase the diversity and abundance of soil microbial communities, and increase the proportion of beneficial microbes such as *Nocardia*, *Blastomonas*, *Bradyrhizobium*, and *Penicillium.* It suggests that marigold rotation has a positive effect on the soil microecological environment. However, the impact of tobacco-marigold rotation on soil physicochemical properties, enzymatic activity, and functional potential of soil microbial communities is unclear and needs further study.

## Supplementary Information

Supplementary Information 1.

Supplementary Information 2.

Supplementary Information 3.

Supplementary Information 4.

Supplementary Information 5.

Supplementary Information 6.

Supplementary Information 7.

Supplementary Information 8.

Supplementary Information 9.

Supplementary Information 10.

Supplementary Information 11.

Supplementary Information 12.

Supplementary Information 13.

Supplementary Information 14.

Supplementary Information 15.

Supplementary Information 16.

Supplementary Information 17.

Supplementary Information 18.

Supplementary Information 19.

Supplementary Information 20.

Supplementary Information 21.

Supplementary Information 22.

Supplementary Information 23.

Supplementary Information 24.

## Data Availability

All data generated or analysed during this study are included in this published article [and its supplementary information files].
